# Impact of Metastasis-directed Therapy Guided by Different PET/CT Radiotracers on Distant and Local Disease Control in Oligorecurrent Hormone-sensitive Prostate Cancer: A Secondary Analysis of the PRECISE-MDT Study

**DOI:** 10.1148/rycan.240150

**Published:** 2025-05-16

**Authors:** Francesco Lanfranchi, Liliana Belgioia, Domenico Albano, Luca Triggiani, Flavia Linguanti, Luca Urso, Rosario Mazzola, Alessio Rizzo, Elisa D’Angelo, Francesco Dondi, Eneida Mataj, Gloria Pedersoli, Elisabetta Maria Abenavoli, Luca Vaggelli, Beatrice Detti, Naima Ortolan, Antonio Malorgio, Alessia Guarneri, Federico Garrou, Matilde Fiorini, Serena Grimaldi, Pietro Ghedini, Giuseppe Carlo Iorio, Antonella Iudicello, Guido Rovera, Giuseppe Fornarini, Diego Bongiovanni, Michela Marcenaro, Filippo Maria Pazienza, Giorgia Timon, Matteo Salgarello, Manuela Racca, Mirco Bartolomei, Stefano Panareo, Umberto Ricardi, Francesco Bertagna, Filippo Alongi, Salvina Barra, Silvia Morbelli, Gianmario Sambuceti, Matteo Bauckneht

**Affiliations:** ^1^Department of Health Sciences (DISSAL), University of Genoa, Genoa, Italy; ^2^Department of Radiotherapy, IRCCS Ospedale Policlinico San Martino, Genoa, Italy; ^3^Department of Nuclear Medicine, ASST Spedali Civili di Brescia, University of Brescia, Brescia, Italy; ^4^Department of Radiation Oncology, ASST Spedali Civili di Brescia, University of Brescia, Brescia, Italy; ^5^Department of Nuclear Medicine, Careggi University Hospital, Florence, Italy; ^6^Department of Nuclear Medicine, Ospedale San Donato, Arezzo, Italy; ^7^Nuclear Medicine, Oncological Medical and Specialist Department, University Hospital of Ferrara, Ferrara, Italy; ^8^Advanced Radiation Oncology Department, IRCCS Sacro Cuore Don Calabria Hospital, Cancer Care Center, Negrar, Verona, Italy; ^9^Department of Nuclear Medicine, Candiolo Cancer Institute, FPO–IRCCS, Turin, Italy; ^10^Department of Radiation Oncology, University Hospital of Modena, Modena, Italy; ^11^Department of Radiation Oncology, Careggi University Hospital, Florence, Italy; ^12^Department of Radiotherapy, University Hospital of Ferrara, Ferrara, Italy; ^13^Department of Radiation Oncology, Candiolo Cancer Institute, FPO–IRCCS, Turin, Italy; ^14^Department of Nuclear Medicine, AOU Città della Salute e della Scienza di Torino, University of Turin, Turin, Italy; ^15^Department of Nuclear Medicine, Oncology and Hematology, University Hospital of Modena, Modena, Italy; ^16^Department of Oncology, Radiation Oncology, University of Turin, Turin, Italy; ^17^Department of Medical Oncology 1, IRCCS Ospedale Policlinico San Martino, Genoa, Italy; ^18^Department of Nuclear Medicine, IRCCS Ospedale Sacro Cuore Don Calabria, Negrar, Italy; ^19^Department of Nuclear Medicine, IRCCS Ospedale Policlinico San Martino and University of Genoa, Largo Rosanna Benzi 10, 16132 Genoa, Italy; ^20^University of Brescia, Brescia, Italy

**Keywords:** Radiation Therapy, Oncology, Urinary, Prostate, PET/CT

## Abstract

Prospective trials suggest that metastasis-directed therapy (MDT) is an effective treatment for patients with oligometastatic prostate cancer (PCa). Gallium 68 (^68^Ga) prostate-specific membrane antigen (PSMA)-11 PET/CT-guided MDT seems to improve the oncologic outcome in these patients compared with fluorine 18 (^18^F)-fluorocholine and ^18^F-PSMA-1007 PET/CT-guided MDT, but the effects in terms of local or distant disease control remain unclear. Thus, the present subanalysis of the PRECISE-MDT study analyzed patients with hormone-sensitive PCa who underwent MDT guided by PET/CT for nodal or bone oligorecurrent disease and were restaged with the same imaging modality in case of biochemical recurrence after MDT. Among 340 lesions detected in 241 male patients (median age, 74 [IQR, 9] years), ^18^F-fluorocholine, ^68^Ga-PSMA-11, and ^18^F-PSMA-1007 PET/CT-guided MDT was performed in 179, 81, and 80 lesions, respectively. At restaging imaging, the PET/CT imaging modality used to guide MDT was not significantly associated with local recurrence-free survival (LRFS), with median LRFS not reached for ^68^Ga-PSMA-11 PET/CT, ^18^F-PSMA-11 PET/CT, and ^18^F-fluorocholine PET/CT (*P* = .73). However, the detection rate of a new metastasis was significantly higher if MDT was guided by ^18^F-fluorocholine PET/CT (119 of 179 lesions, 66.5%) compared with ^68^Ga-PSMA-11 or ^18^F-PSMA-1007 PET/CT (23 of 81 lesions, 28%, and 27 of 80, 34%, respectively; *P* < .001 for both). Moreover, MDT guided by ^68^Ga-PSMA-11 PET/CT led to an improved median metastasis-free survival (MFS) (not reached) compared with ^18^F-PSMA-1007 (median MFS, 24.9 months; *P* < .001) or ^18^F-fluorocholine PET/CT (median MFS, 18 months; *P* < .001). These findings suggest that using different PET/CT imaging modalities to guide MDT might impact the distant disease control in this clinical scenario.

**Keywords:** Radiation Therapy, Oncology, Urinary, Prostate, PET/CT

*Supplemental material is available for this article.*

Published under a CC BY 4.0 license.

**Summary**

Metastasis-directed therapy guided by gallium 68 (^68^Ga) prostate-specific membrane antigen (PSMA)-11 PET/CT improved metastasis-free survival but not local recurrence-free survival compared with fluorine 18 (^18^F)-PSMA-1007 and ^18^F-fluorocholine PET/CT in patients with nodal or bone oligorecurrent prostate cancer.

**Key Points**

■ In patients with oligorecurrent prostate cancer, metastasis-directed therapy (MDT) guided by gallium 68 (^68^Ga) prostate-specific membrane antigen (PSMA)-11 PET/CT led to improved distant disease control (median metastasis-free survival [MFS] not reached) compared with fluorine 18 (^18^F) PSMA-1007 (median MFS, 24.9 months; *P* < .001) and ^18^F-fluorocholine PET/CT (median MFS, 18 months; *P* < .001).
■ Improved metastasis-free survival with ^68^Ga-PSMA-11 PET/CT-guided MDT compared with the other two PET/CT modalities was also observed in separate analyses of nodal and bone lesions (*P* < .001 for both).
■ No evidence of differences in local disease control (median local recurrence-free survival [LRFS]) was detected between the three PET/CT modalities as the guide for MDT (median LRFS was not reached for ^68^Ga-PSMA-11 PET/CT, ^18^F-PSMA-11 PET/CT, or ^18^F-fluorocholine PET/CT; *P* = .73 for all lesions, *P* = .52 for nodal metastases, and *P* = .69 for bone metastases).

## Introduction

Prospective trials have demonstrated that metastasis-directed therapy (MDT) using stereotactic body radiation therapy improves oncologic outcomes in patients with oligometastatic prostate cancer (PCa) (1–3). However, the optimal imaging modality to guide MDT remains to be established. A few studies have compared the prognostic value of different radiotracers in PET/CT imaging for this purpose (4–9). In most cases, MDT guided by gallium 68 (^68^Ga) prostate-specific membrane antigen (PSMA)-11 PET/CT demonstrated favorable effects on androgen deprivation therapy–free survival and progression-free survival (PFS) compared with MDT guided by choline PET/CT. Moreover, the multicenter PRECISE-MDT study showed a favorable effect of PSMA PET/CT compared with choline PET/CT in terms of time to treatment change due to polymetastatic conversion (PFS2) and overall survival in patients with oligorecurrent PCa (9). Additionally, the study reported that ^68^Ga-PSMA-11 PET/CT-guided MDT could improve PFS and PFS2 even compared with fluorine 18 (^18^F)-PSMA-1007 PET/CT. Nevertheless, the variability in patient characteristics and outcome measures complicates the identification of the determinants of this finding. Specifically, the effects of different imaging modalities on local or distant disease control have yet to be explored.

Therefore, the present study aimed to verify at a lesion-based level whether different PET/CT radiotracers could influence the rate of local or distant disease control in patients with oligorecurrent PCa undergoing imaging-guided MDT.

## Materials and Methods

### Study Sample and Data Collection

This study was a subanalysis of the previously published, retrospective PRECISE-MDT study (9). It adhered to Declaration of Helsinki guidelines and received approval from the local ethical committee (Regional Ethical Committee of Regione Liguria, registration number 5/2023-DB id 12914). Written informed consent was obtained from included patients.

This subanalysis exclusively focused on patients with oligorecurrent hormone-sensitive PCa from the PRECISE-MDT study cohort (the flow diagram for patients’ selection is displayed in Fig S1). Patients underwent MDT guided by next-generation imaging modalities (^18^F-fluorocholine, ^68^Ga-PSMA-11, or ^18^F-PSMA-1007 PET/CT) to detect up to five nodal or bone PCa metastases at eight tertiary-level cancer centers between July 2012 and May 2023. Inclusion was restricted to patients restaged with the same imaging modality as that used during the initial MDT, in the event of a subsequent biochemical recurrence. Patients with more than five metastases were not included in the study, as they were treated with systemic therapies.

### Imaging-guided MDT and Follow-up

PET/CT was performed following current guidelines (10,11). The different scanners used at each center are detailed in Table S1. Imaging-guided MDT, either through intensity-modulated radiation therapy or volumetric-modulated arc therapy, was administered to all patients following current international guidelines and recommendations for good clinical practice (12). The follow-up protocol started from the end of the administration of MDT until the detection of any events or the last clinical evaluation, and it was carried out according to the institutional protocols of each center, including clinical evaluations and prostate-specific antigen (PSA) blood tests every 3–6 months. In cases of biochemical recurrence, patients underwent PET/CT for restaging in alignment with current guidelines (10).

### Statistical Analysis

Data distribution was assessed with the Kolmogorov-Smirnov test. Categorical variables were compared with the χ^2^ test or Fisher exact test. Continuous variables were compared with the Kruskal-Wallis test, and post hoc analyses were performed using the Mann-Whitney test, applying the Bonferroni correction for multiple comparisons. A per-lesion approach was adopted to assess clinical, biochemical, imaging, and treatment predictors of local recurrence-free survival (LRFS) and metastasis-free survival (MFS). LRFS was defined as the interval between the administration of MDT and PET/CT restaging in which persistent or increased tracer uptake was detected compared with the baseline in the same lesion treated with MDT. MFS was defined as the time from MDT to the detection of a new metastatic site at restaging of PET/CT. If patients received MDT on multiple lesions, local events were counted for the specific metastases treated and recurred, while distant events were attributed to all lesions. A multivariable Cox regression model was built to determine the association of LRFS and MFS with clinical, biochemical, imaging, and treatment parameters. Hazard ratios (HRs) were reported with 95% CIs and *P* values. The Kaplan-Meier method (log-rank test) was applied to explore differences in LRFS and MFS in patients according to different imaging modalities used to guide MDT.

A sensitivity analysis was performed with the leave-one-out approach, sequentially excluding data from each participating center to assess its influence on the main results.

Statistical analysis was conducted using SPSS version 26 (IBM SPSS Statistics for Windows, Armonk) and MedCalc version 19.4 (MedCalc Software), and significance was set at *P* < .05.

## Results

### Clinical, Treatment, and Follow-up Characteristics

Pre-MDT clinical and biochemical characteristics of the 241 patients (all male) included in this study are summarized in Table 1. Continuous and dichotomous variables are reported as medians and IQRs (calculated as quartile 3 minus quartile 1) as nonnormally distributed and absolute frequencies and percentages, respectively. For the considered parameters, values were available for all patients, thus avoiding missing data. At the time of PCa diagnosis, 63.1% (152 of 241) of patients were classified as having American Joint Committee on Cancer stage III disease, while 11.6% (28 of 241) had stage IV disease. Lesions with International Society of Urological Pathology grades 1–5 were present in 37, 59, 57, 35, and 53 of the 241 patients, respectively. Surgery was the primary treatment in 201 of the 241 patients, with 35 of 241 receiving radical radiation therapy (with or without androgen-deprivation therapy) and five of 241 receiving medical therapy alone.

**Table 1:**
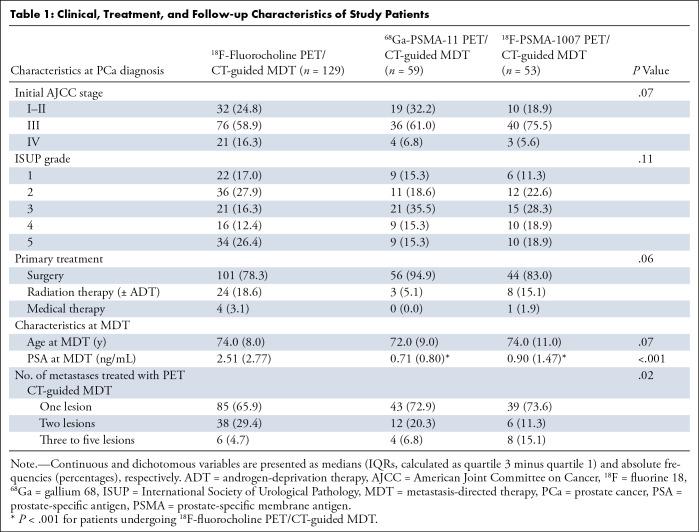
Clinical, Treatment, and Follow-up Characteristics of Study Patients

At the time of MDT, the median age of the sample was 74 years (range: 53–90 years). ^18^F-fluorocholine, ^68^Ga-PSMA-11, and ^18^F-PSMA-1007 PET/CT were used to guide MDT in 129, 59, and 53 of the 241 patients, respectively. The median PSA levels were significantly lower in patients who underwent PSMA PET/CT-guided MDT (0.71 ng/mL and 0.90 ng/mL in the ^68^Ga-PSMA-11 and ^18^F-PSMA-1007 groups, respectively) compared with the choline PET/CT-guided MDT group (2.51 ng/mL; *P* < .001 compared with both). The percentage of patients with three to five metastases treated with MDT was significantly higher in the ^18^F-PSMA-1007 group (15%, eight of 53) compared with the other two groups (^68^Ga-PSMA-11: 6.8%, four of 59; choline: 4.7%, six of 129; *P* = .02 for both).

Treatment parameters and follow-up data at the per-lesion analysis are displayed in Table 2. There were 340 lesions identified at PET/CT and treated with MDT; 84 of the 340 were bone metastases, and 256 of the 340 were lymph node localizations. The frequency of bone localizations was significantly more pronounced in patients who underwent ^18^F-PSMA-1007 PET/CT (32 of 80, 40%) compared with the other two groups (^68^Ga-PSMA-11: 14 of 81, 17%; choline: 38 of 179, 21.2%; *P* = .001 for both). Systemic treatments in addition to MDT were administered less frequently in patients who underwent ^18^F-fluorocholine or ^18^F-PSMA-1007 PET/CT (53 of 179, 29.6%, and 18 of 80, 22.5%, respectively) compared with patients who underwent ^68^Ga-PSMA-11 PET/CT (42 of 81, 51.9%; *P* < .001 compared with both).

**Table 2:**
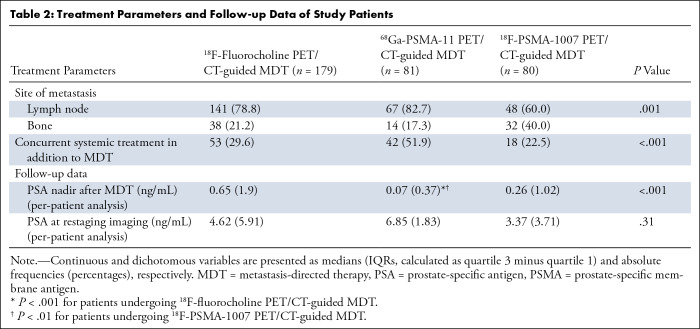
Treatment Parameters and Follow-up Data of Study Patients

After undergoing MDT treatment, patients were followed up for a median interval of 33.63 months (range: 6.46–116.70 months). For those patients without events during follow-up, the median duration was 24.45 months (range: 7.03–82.43 months).

The median PSA nadir was significantly lower in the ^68^Ga-PSMA-11 PET/CT-guided MDT group (0.07 ng/mL) compared with the ^18^F-PSMA-1007 (0.26 ng/mL; *P* = .007) or ^18^F-fluorocholine PET/CT-guided MDT (0.65 ng/mL; *P* < .001) groups. In contrast, there was no evidence of a difference between the three groups in PSA levels at restaging imaging (*P* = .31).

During the entire follow-up time, the cumulative number of local and distant recurrence events in the 340 lesions was 30 (8.8%) and 169 (49.7%), respectively. Local relapse occurred in 18 of the 179 lesions in the ^18^F-fluorocholine group, six of the 81 in the ^68^Ga-PSMA-11 group, and six of the 80 in the ^18^F-PSMA-1007 group. The detection of a new metastatic localization occurred in 119 of 179 (66.5%), 23 of 81 (28%), and 27 of 80 (34%) cases when MDT was guided by ^18^F-fluorocholine, ^68^Ga-PSMA-11, or ^18^F-PSMA-1007 PET/CT, respectively.

### Local Disease Control

At multivariable analysis, bone metastatic lesions showed significant prediction of a higher risk of local recurrence after MDT (HR: 3.37 [95% CI: 1.02, 11.1]; *P* = .046), while concurrent systemic treatment in addition to MDT predicted higher risk of local disease control (HR: 0.37 [95% CI: 0.14, 0.97]; *P* = .04) (Table 3).

**Table 3:**
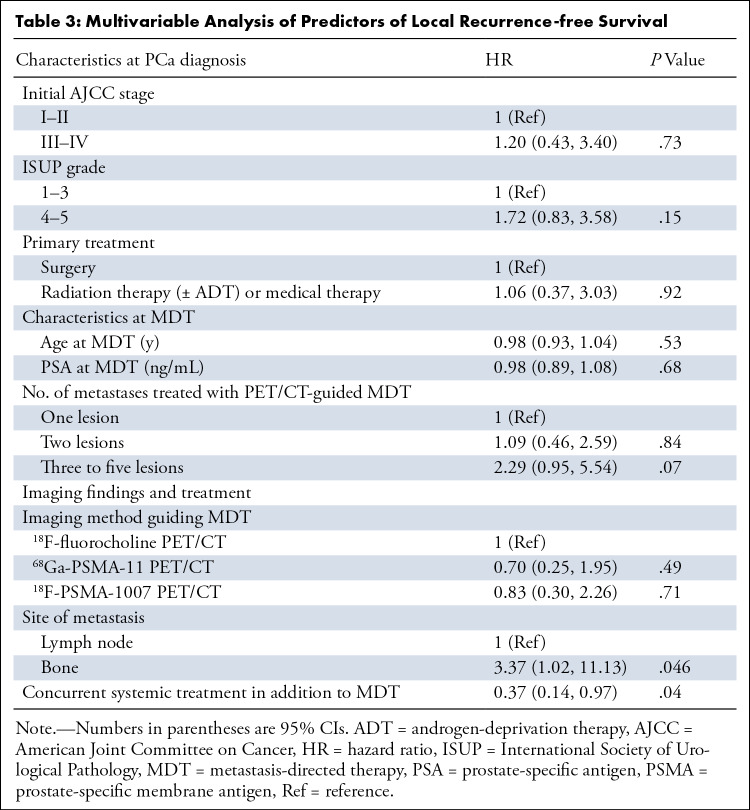
Multivariable Analysis of Predictors of Local Recurrence-free Survival

**Figure 1:**
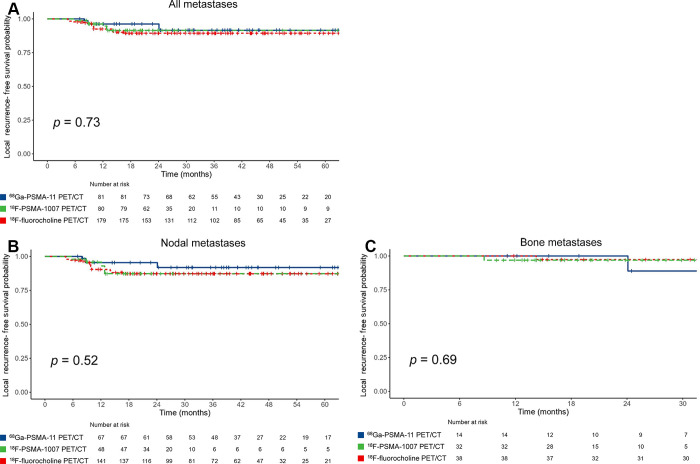
Kaplan-Meier curves (log-rank test) show local recurrence-free survival according to the PET/CT radiopharmaceutical used to guide metastasis-directed therapy in **(A)** all lesions and **(B)** nodal or **(C)** bone metastases. ^18^F = fluorine 18, ^68^Ga = gallium 68, PSMA = prostate-specific membrane antigen.

In the entire study sample, as well as in the ^18^F-fluorocholine, ^68^Ga-PSMA-11, and ^18^F-PSMA-1007 PET/CT subgroups, the median LRFS was not reached. Kaplan-Meier curves revealed no evidence of differences in median LRFS among the three subgroups (*P* = .73) (Fig 1A), and the sensitivity analysis confirmed this result (Fig S2). Similarly, there was no evidence of a difference in median LRFS among subgroups when nodal and bone lesions were analyzed separately (*P* = .52 and *P* = .69, respectively) (Fig 1B and 1C, respectively).

### Distant Disease Control

In the multivariable analysis, significant predictors of unfavorable MFS included the use of ^18^F-fluorocholine compared with ^18^F-PSMA-1007 (HR: 0.44 [95% CI: 0.28, 0.71]; *P* = .001) or ^68^Ga-PSMA-11 (HR: 0.30 [95% CI: 0.19, 0.47]; *P* < .001) as the guide for MDT, the presence of three to five metastatic lesions (HR: 1.65 [95% CI: 1.06, 2.59]; *P* = .03), and the absence of concurrent systemic treatment in addition to MDT (when androgen-deprivation therapy was added: HR: 0.42 [95% CI: 0.29, 0.61]; *P* < .001) (Table 4).

**Table 4:**
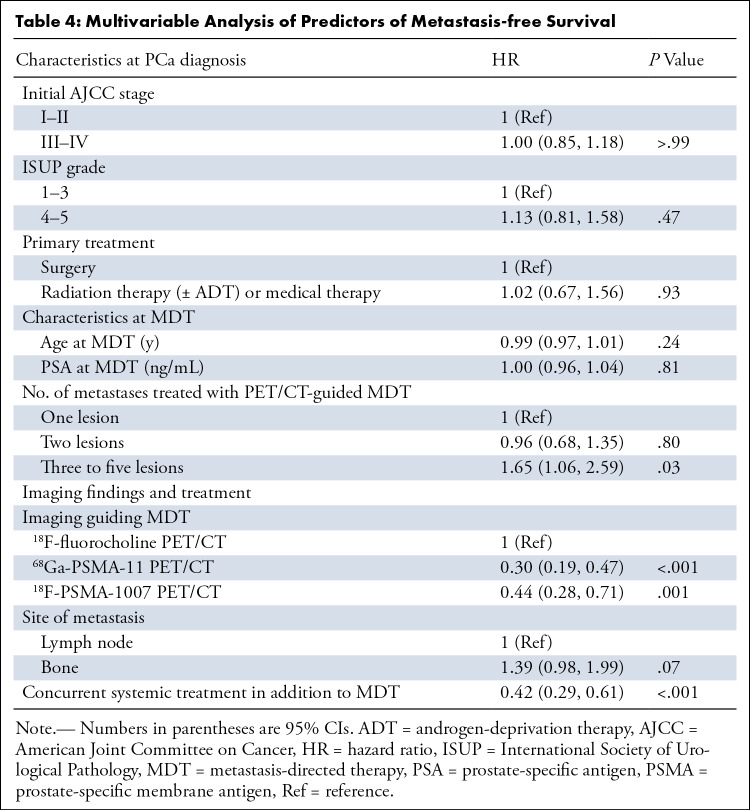
Multivariable Analysis of Predictors of Metastasis-free Survival

**Figure 2:**
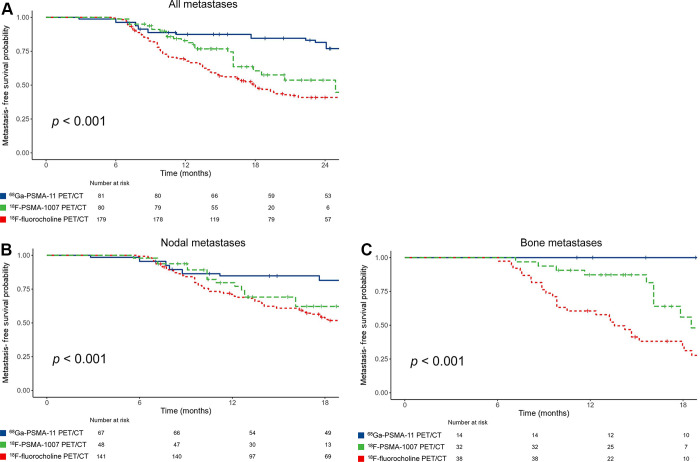
Kaplan-Meier curves (log-rank test) show metastasis-free survival according to the PET/CT radiopharmaceutical used to guide metastasis-directed therapy in **(A)** all lesions and **(B)** nodal or **(C)** bone metastases. ^18^F = fluorine 18, ^68^Ga = gallium 68, PSMA = prostate-specific membrane antigen.

The median MFS for the entire patient sample was 25.93 months (95% CI: 16.89, 34.98). As shown in Figure 2A, MDT guided by ^68^Ga-PSMA-11 PET/CT led to an improved median MFS (not reached) compared with ^18^F-PSMA-1007-guided MDT (24.9 months [95% CI: 14.8, 34.9]; *P* < .001), which, in turn, led to an improved median MFS compared with ^18^F-fluorocholine PET/CT-guided MDT (18 months [95% CI: 15.1, 20.9]; *P* < .001). This result was confirmed at the sensitivity analysis (Fig S3). This outcome was also observed at Kaplan-Meier analyses for both nodal and bone lesions (*P* < .001 for both) (Fig 2B and 2C, respectively).

**Figure 3:**
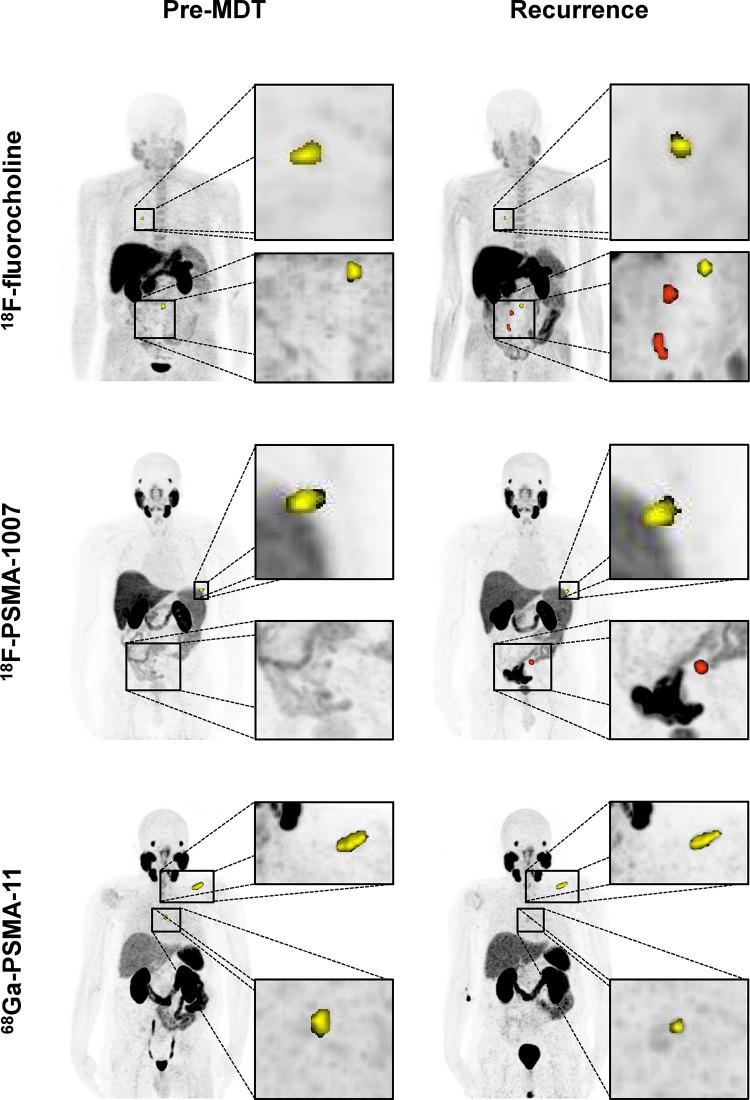
Representative maximum-intensity projections of PET/CT scans obtained with different radiopharmaceuticals before metastasis-directed therapy (MDT) and at the time of imaging recurrence. Scans obtained before MDT (left) show lesions that underwent MDT (yellow), while follow-up images (right) display both local recurrences (yellow) and new metastases (red). ^18^F = fluorine 18, ^68^Ga = gallium 68, PSMA = prostate-specific membrane antigen.

Representative images of local and distant recurrences after PET/CT-guided MDT with the three different radiotracers are shown in Figure 3.

## Discussion

The impact of different PET/CT radiopharmaceuticals as the guide for MDT in terms of local or distant disease control in patients with oligorecurrent hormone-sensitive PCa is still unclear. Among 340 lesions detected in 241 male patients included in the present subanalysis of the PRECISE-MDT study, MDT guided by ^68^Ga-PSMA-11 PET/CT led to an improved median MFS (not reached) compared with ^18^F-PSMA-1007 (24.9 months; *P* < .001) or ^18^F-fluorocholine PET/CT (18 months; *P* < .001). On the contrary, the PET/CT modality used to guide MDT was not significantly associated with local disease control, with median LRFS not reached for the ^68^Ga-PSMA-11 PET/CT, ^18^F-PSMA-11 PET/CT, or ^18^F-fluorocholine PET/CT groups (*P* = .73).

The absence of a significant association between the imaging modalities used to guide MDT and local disease control is consistent with the existing literature. Indeed, previous studies have highlighted that choline PET/CT is specific in identifying PCa lesions, even when compared with PSMA imaging, especially in the biochemical recurrence setting (13,14). However, choline PET/CT exhibits lower sensitivity and detection rates compared with both ^68^Ga-PSMA-11 and ^18^F-PSMA-1007 PET/CT, especially in patients with lower PSA levels (12–17). These differences likely contribute to the improved MFS observed in patients undergoing MDT guided by PSMA imaging compared with ^18^F-fluorocholine PET/CT, suggesting that using a more sensitive imaging modality for MDT guidance can enhance control of distant recurrences.

Further analysis has revealed a hierarchy among PSMA-targeted tracers, with ^68^Ga-PSMA-11 PET/CT demonstrating improved MFS compared with ^18^F-PSMA-1007 when guiding MDT. The optimal PSMA-targeted ligand for clinical use has yet to be determined (17). However, ^18^F-PSMA-1007 shows higher uptake in nodal and bone lesions of benign origin (with the latter referred to as unspecific bone uptakes) compared with ^68^Ga-PSMA-11 (18,19). This higher uptake may complicate image interpretation for less experienced PET readers (20). On the one hand, true bone metastases may have been misinterpreted as unspecific bone uptakes, potentially leading to undertreatment and inaccurate MDT targeting. On the other hand, nonspecific uptakes during follow-up scans could have been mistakenly identified as distant recurrences. These factors may account for the improved MFS observed with ^68^Ga-PSMA-11 PET/CT-guided MDT.

Collectively, our findings corroborate previous evidence that the choice of PET radiotracer influences clinical outcomes in patients undergoing MDT for oligorecurrent hormone-sensitive PCa (9). However, this study uniquely explores the differences in rates of local versus distant control, suggesting that the latter may be a key determinant of oncologic outcomes.

Our study had limitations. First, it lacked a prospective and randomized design, so future studies must confirm our findings. Nonetheless, it was a deliberate decision by the authors to evaluate current clinical practice from a real-world perspective. For the same reason, in some cases of biochemical recurrence after MDT, patients were either not restaged or restaged using a different imaging modality than the one initially guiding MDT. These cases were excluded from our subanalysis, which may have introduced selection bias into our sample. Furthermore, some clinical differences potentially influencing the clinical outcome have been detected among the three groups of patients. However, all these characteristics were included in the multivariable model, and the imaging modality adopted to guide MDT showed independent prognostic power. Additionally, the pronounced differences between choline and PSMA imaging in patients with lower PSA levels could not be fully explored due to our sample’s limited size, nor could we assess replicability in the castration-resistant setting. Last, the limited number of events in our sample precluded an analysis of overall survival as a study outcome.

In conclusion, the use of different next-generation imaging modalities to guide MDT in patients with oligorecurrent hormone-sensitive PCa might impact clinical outcomes by influencing distant disease control. In our retrospective secondary analysis, ^68^Ga-PSMA-11 PET/CT seems to be the preferable imaging tool over ^18^F-fluorocholine and even ^18^F-PSMA-1007 PET/CT. Further studies with larger samples and a prospective design are necessary to confirm these results.
